# An Unusual Presentation of Orbital Compartment Syndrome: A Case Report

**DOI:** 10.5811/cpcem.46993

**Published:** 2025-08-26

**Authors:** Jillian Rosenblum

**Affiliations:** Alta Bates Summit Medical Center, Emergency Department, Oakland, California

**Keywords:** orbital compartment syndrome, Saturday night retinopathy, lateral canthotomy, case report

## Abstract

**Introduction:**

Orbital compartment syndrome (OCS) is a rare but high-morbidity emergency requiring prompt recognition and management.

**Case Report:**

We present a case of a man who developed OCS from external compression of the globe while lying in a prone position. Initially obtunded and unable to provide any history, the patient exhibited anisocoria, which later progressed to severe chemosis and proptosis. Intraocular pressure reached nearly 100 millimeters of mercury, improving immediately after emergent lateral canthotomy with cantholysis. His course was complicated by ipsilateral limb compartment syndrome and worsening renal failure requiring dialysis.

**Conclusion:**

This case highlights the critical role emergency physicians play in the rapid diagnosis and treatment of orbital compartment syndrome.

## INTRODUCTION

Orbital compartment syndrome (OCS) is an ophthalmologic emergency in which a sudden increase in pressure within the orbit compromises blood flow to the optic nerve and retina, risking permanent vision loss. This case presents a rare etiology of OCS caused by external compression of the globe due to a prolonged, drug-induced downtime in the prone position. An ischemic reperfusion injury occurred once the patient remained in a supine position in the emergency department (ED). This differs from the typical cause of OCS, which is a retrobulbar hematoma following blunt facial trauma. The patient’s obtunded state, coupled with a lack of the typical finding of a retrobulbar hematoma on computed tomography (CT) of the brain, posed a diagnostic challenge. This case emphasizes the need for emergency physicians to remain vigilant in the rapid diagnosis and treatment of OCS, particularly in atraumatic cases and when the patient cannot report history or symptoms.

## CASE REPORT

A 34-year-old man with a prior history of schizophrenia and substance use disorder presented to the ED with altered mental status, found unresponsive on the sidewalk with unknown downtime. He was given naloxone in the field with no improvement in mental status. On arrival, he was moaning incomprehensibly and not answering questions or following commands. He was found to be hypothermic, tachycardic, tachypneic, and oxygen saturation was 72% on room air. Physical examination revealed anisocoria, with the right pupil two millimeters larger than the left and non-reactive to light. His point-of-care glucose revealed severe hypoglycemia. He was given dextrose with no improvement in mental status, followed by intubation and placement of warming blankets.

Laboratory studies were notable for a leukocytosis with left shift, hyperkalemia with a potassium of 6.5 millimoles per liter (mmol/L) (reference range: 3.5–5.1 mmol/L), an elevated creatinine of 2.53 milligrams per deciliter (mg/dL) (0.50–1.30 mg/dL), elevated liver function tests with aspartate aminotransferase 805 units per liter (U/L) (0–37 U/L) and alanine transaminase 207 U/L (0–60 U/L), a high sensitivity troponin slightly over 2,000 nanograms (ng)/L (0–76 ng/L), a creatine kinase of 46,316 U/L (39–308 U/L), a lactic acid of 12.8 mmol/L (0.4–2.0 mmol/L), pH on arterial blood gas of 7.1 (7.35–7.45), negative serum alcohol level, and urine drug screen positive for amphetamines, cannabinoids, and fentanyl.

Electrocardiogram demonstrated no ST-segment elevation or arrhythmia. Initial imaging included a chest radiograph and computed tomography (CT) of the brain without contrast, which were significant for a right upper lobe infiltrate and a new, hypoattenuating lesion in the basal ganglia concerning for an acute infarction. He was promptly treated with sodium bicarbonate, calcium gluconate, lactated Ringer, broad spectrum intravenous antibiotics, and a norepinephrine infusion for mean arterial pressures consistently below 65 millimeters of mercury (mm Hg). He was placed on a propofol infusion for sedation.

While awaiting transportation to the intensive care unit, he developed new right-sided proptosis and chemosis. Radiology was called to re-evaluate the CT of the brain with special attention paid to his right orbit. It was then noticed that he had diffusely thickened right extraocular motor muscles (Image). Intraocular pressures were checked and found to be elevated to 97 mm Hg on the right and normal on the left (< 21 mm Hg). Emergent right lateral canthotomy with cantholysis was performed with immediate improvement in right intraocular pressure.

*CPC-EM Capsule*

What do we already know about this clinical entity?

*Orbital compartment syndrome (OCS) is a rare but vision-threatening emergency that usually occurs in the setting of blunt facial trauma*.

What makes this presentation of disease reportable?

*This case describes an atraumatic cause of OCS in a patient with a prolonged period of unconsciousness in the prone position*.

What is the major learning point?

*OCS can occur in the absence of trauma and must be considered in obtunded patients with proptosis or anisocoria*.

How might this improve emergency medicine practice?

*This case raises awareness of atypical OCS presentations, enabling clinicians to improve their diagnostic vigilance and ultimately prevent permanent vision loss*.

The following day the patient’s clinical course was complicated by right lower extremity compartment syndrome requiring open fasciotomy of the upper and lower leg compartments. His renal function worsened with worsening rhabdomyolysis, hyperkalemia, and lactic acidosis requiring hemodialysis. By day 10, he was extubated and discharged in stable condition, with the need for ongoing hemodialysis as an outpatient and a poor prognosis for return of vision. He was instructed to follow up with plastic surgery for future skin grafting of his right leg, with cardiology for suspected drug-induced cardiomyopathy, and with ophthalmology.

## DISCUSSION

Orbital compartment syndrome is rare, occurring in less than 1% of ED presentations involving facial trauma.^1^ It most often occurs due to retrobulbar hemorrhage following blunt facial trauma.^1^ Prompt recognition and treatment are crucial to prevent rapid and irreversible vision loss. Making the diagnosis can be particularly challenging when a patient is obtunded and cannot provide history or report symptoms of eye pain or vision loss. Orbital compartment syndrome is a clinical diagnosis made based on signs, symptoms, and intraocular pressure measurements.^2^ Clinical signs can include an afferent pupillary defect, proptosis, tense periorbital edema, limited extraocular movements, and a firm globe due to elevated intraocular pressure.^3^ Optimal outcomes are achieved if treatment is initiated within 90 minutes of onset.^4^

In our case, the presumed cause of OCS was external compression of the globe in the setting of prolonged, drug-induced downtime in a prone position, which caused an ischemic reperfusion injury once the patient remained supine. This is a paradoxical phenomenon in which the restoration of blood flow to ischemic tissue triggers an inflammatory response that further exacerbates tissue damage. The ophthalmology literature refers to this entity as “Saturday night retinopathy.”^5^ No treatment will positively affect visual prognosis given that by the time of patient presentation, the ischemic insult from sustained external compression has usually already occurred.^3^ Vision loss is typically severe and permanent.

On the other hand, promptly performing a lateral canthotomy and cantholysis for traumatic cases of OCS is highly likely to be successful, with alleviation of pressure in the orbital compartment occurring between 68–79% of the time.^6^ The key is rapid diagnosis and initiation of management, as permanent vision loss can be prevented if performed within two hours of onset.^7^ If OCS is not relieved by canthotomy and cantholysis, the patient may need open orbitotomy and bony orbital decompression in the operating room.^4^ Systemic treatments, such as diuretics like acetazolamide and mannitol, have no role in management of OCS.^4^ Care measures do include adequate pain and blood pressure control, elevating the head of the bed, and minimizing Valsalva maneuver by giving antiemetics, decongestants, and stool softeners.^4^ Complications from a lateral canthotomy are relatively rare when performed correctly but can include iatrogenic injury to the globe, the lacrimal gland and artery, and the eyelids. Patients should be treated with aggressive eye lubrication until outpatient follow-up with an ophthalmologist, given the risk of exposure keratopathy from the lateral canthus deformity.^4^

## CONCLUSION

This case underscores the emergency physician’s vital role in the rapid diagnosis and management of orbital compartment syndrome. Lateral canthotomy with cantholysis must be performed emergently, often before an ophthalmologist is available, to prevent permanent vision loss. Emergency physicians must recognize and treat OCS expeditiously, particularly in obtunded patients where clinical signs can be subtle.

## Figures and Tables

**Image:**
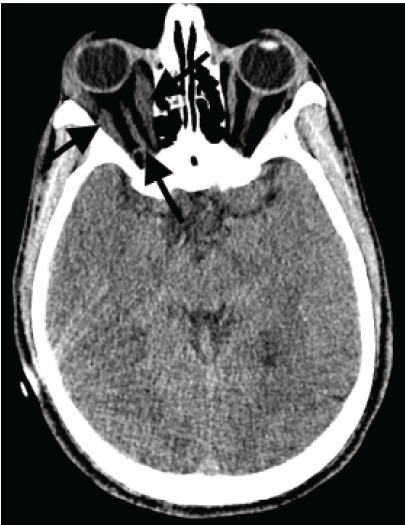
Computed tomography of the brain with thickened right extraocular muscles in a patient with orbital compartment syndrome (black arrows)
